# Sex-specific interactions between stress axis and redox balance are associated with internalizing symptoms and brain white matter microstructure in adolescents

**DOI:** 10.1038/s41398-023-02728-4

**Published:** 2024-01-17

**Authors:** Zoé Schilliger, Yasser Alemán-Gómez, Mariana Magnus Smith, Zeynep Celen, Ben Meuleman, Pierre-Alain Binz, Pascal Steullet, Kim Q. Do, Philippe Conus, Arnaud Merglen, Camille Piguet, Daniella Dwir, Paul Klauser

**Affiliations:** 1 Center for Psychiatric Neuroscience, Department of Psychiatry, Lausanne University Hospital and University of Lausanne, Lausanne, Switzerland; 2 Service of Child and Adolescent Psychiatry, Department of Psychiatry, Lausanne University Hospital and University of Lausanne, Lausanne, Switzerland; 3 Connectomics Lab, Department of Radiology, Lausanne University Hospital and University of Lausanne, Lausanne, Switzerland; 4 Division of General Pediatrics, Geneva University Hospitals and Faculty of Medicine, University of Geneva, Geneva, Switzerland; 5 Department of Psychiatry, Faculty of Medicine, University of Geneva, Geneva, Switzerland; 6 Service of Clinical Chemistry, Lausanne University Hospital and University of Lausanne, 1011 Lausanne, Switzerland; 7 Service of General Psychiatry, Department of Psychiatry, Lausanne University Hospital and University of Lausanne, Lausanne, Switzerland

**Keywords:** Biomarkers, Molecular neuroscience

## Abstract

Adolescence is marked by the maturation of systems involved in emotional regulation and by an increased risk for internalizing disorders (anxiety/depression), especially in females. Hypothalamic-pituitary-adrenal (HPA)-axis function and redox homeostasis (balance between reactive oxygen species and antioxidants) have both been associated with internalizing disorders and may represent critical factors for the development of brain networks of emotional regulation. However, sex-specific interactions between these factors and internalizing symptoms and their link with brain maturation remain unexplored. We investigated in a cohort of adolescents aged 13–15 from the general population (*n* = 69) whether sex-differences in internalizing symptoms were associated with the glutathione (GSH)-redox cycle homeostasis and HPA-axis function and if these parameters were associated with brain white matter microstructure development. Female adolescents displayed higher levels of internalizing symptoms, GSH-peroxidase (GPx) activity and cortisol/11-deoxycortisol ratio than males. There was a strong correlation between GPx and GSH-reductase (Gred) activities in females only. The cortisol/11-deoxycortisol ratio, related to the HPA-axis activity, was associated with internalizing symptoms in both sexes, whereas GPx activity was associated with internalizing symptoms in females specifically. The cortisol/11-deoxycortisol ratio mediated sex-differences in internalizing symptoms and the association between anxiety and GPx activity in females specifically. In females, GPx activity was positively associated with generalized fractional anisotropy in widespread white matter brain regions. We found that higher levels of internalizing symptoms in female adolescents than in males relate to sex-differences in HPA-axis function. In females, our results suggest an important interplay between HPA-axis function and GSH-homeostasis, a parameter strongly associated with brain white matter microstructure.

## Introduction

Adolescence is marked by important brain morphological and functional changes of affect-related systems, such as the corticolimbic network. Among maturational processes, synaptic refinement and axonal myelination in prefrontal and limbic areas may promote adult-like emotion and affect regulation strategies [1]. This protracted period of growth is characterized by a heightened sensitivity to the environment, representing both an opportunity for increased adaptability to positive experiences, but also a higher vulnerability to environmental insults, which may impact developmental trajectories [2]. The period of adolescence also witnesses a steep rise in the incidence of internalizing disorders, encompassing anxiety and affective disorders [3], with a worse outcome compared to adult onset [4, 5]. Importantly, puberty appears to set the stage for a higher susceptibility to stress-related disorders in females, as they display an important increase in internalizing disorders [6, 7], setting the well-established 1:2 male:female ratio of depression observed in adults [8]. Longitudinal imaging studies exploring the dynamic of brain changes during adolescence also indicate sex-specific trajectories regarding volumes [9] and microstructure [10]. Puberty seems to be an important factor underlying sexually divergent trajectories, suggesting that physiological changes associated with sexual maturation, such as the rising levels of gonadal hormones, may play sex-specific roles in brain maturation [11]. However, the biological mechanisms promoting sex-difference in brain maturation trajectories as well as differential vulnerability to internalizing disorders remain poorly understood. Both clinical and preclinical findings indicate that the hypothalamic-pituitary-adrenal (HPA)-axis shows a shift towards heightened reactivity and sexually-dimorphic response along with sexual maturation [12–15]. This surge of heightened stress-reactivity has been postulated to yield increased sensitivity to environmental stress, which could impact the maturation of the corticolimbic network implicated in emotion regulation, lastingly increasing the risk for anxiety and affective disorders [16, 17].

Adolescence is a highly metabolically demanding period [18], rendering the brain potentially sensitive to the imbalance between antioxidant capacity and reactive oxygen species (ROS) leading to oxidative stress (OxS) [19]. Preclinical findings indicate increased vulnerability of the brain to redox dysregulation during peripuberty, compared to later developmental stages [20, 21]. Growing evidence indicates that impaired redox homeostasis, as well as subsequent OxS may be critically involved in the development of anxiety and affective disorders [22–25]. Preclinical findings have evidenced a causal link between brain oxidative damages and the emergence of anxiety and depressive-like behavior [25–28]. On the other hand, multiple evidence indicates that chronic stress promotes OxS in stress-sensitive areas such as the prefrontal cortex (PFC) [29], amygdala [30] and hippocampus [25]. While antioxidants can diminish anxiety-like behavior in rodents [28, 31, 32], the expression of two enzymes associated with the antioxidant glutathione (GSH) system (glyoxalase 1 and GSH reductase 1) in emotion-related brain regions modulates anxiety-like behavior [26]. Noteworthy, mice with a limited capacity to synthetize GSH are more vulnerable to adolescent stress-induced anxiety [20]. As one of the major non-enzymatic antioxidant and redox regulator in the brain, GSH acts largely via the GSH-redox cycle (Supplementary Fig. S1) which involves two GSH-dependent enzymes, GSH peroxidase (GPx) and GSH reductase (Gred). The activity of GPx catalyzes the reduction of peroxides by oxidizing GSH into GSSG, while the activity of Gred reduces GSSG back to GSH. The activity of these two enzymes may reflect GSH-redox cycle regulation and were shown to be altered in patients with anxiety and affective disorders [22, 33–35]. Furthermore, glucocorticoid sensitivity [36] and HPA-axis reactivity [28, 37] are influenced by the oxidoreductive status and were shown to normalize upon antioxidant treatment [28, 38]. On the other hand, high doses and/or long-term exposition to glucocorticoids was shown to repress the antioxidant response in the brain and promote oxidative stress [28, 39–41]. However, to our knowledge, no clinical study has investigated the association between peripheral GSH-redox cycle regulation and markers of the HPA-axis, especially during adolescence. Additionally, animal studies suggest that both glucocorticoids and the GSH-antioxidant balance may play a role in modulating myelin maturation [42, 43]. Several clinical studies report negative association between morning cortisol levels and neuroimaging indices of white matter microstructure such as fractional anisotropy (FA) [44, 45], whereas others report developmental exposure to glucocorticoids and stress to be associated with increased FA [46, 47]. Regarding the role of redox regulation, we previously reported a positive association between central levels of GSH in the prefrontal cortex measured by magnetic resonance spectroscopy, and structural and functional connectivity along the cingulum bundle in healthy young adults, suggesting that redox regulation supported by the GSH-redox cycle may be involved in white matter maturation [42]. Association between GSH and FA measured in the limbic system was also evidenced in young adults, with diagnosis of mood disorder and low hippocampal GSH associated with lower FA in the stria terminalis [48]. Given the important changes in HPA-axis reactivity and sensitivity occurring during adolescence and the greater susceptibility to redox dysregulation conferred by the metabolic changes occurring during this period, we aimed to investigate the associations between the homeostasis of the GSH-redox cycle, and markers of HPA-axis activity in blood, as well as their relationship with internalizing symptoms and brain white matter microstructure in young adolescents. In a well characterized sample of adolescents from the general population, we investigated whether adolescents show sex-differences in (1) the levels of self-rated anxiety and depression symptoms, (2) markers of GSH homeostasis, and if this difference is related to internalizing symptoms severity, (3) markers of the HPA axis activity, and if this difference is related to internalizing symptoms severity, (4) the association between markers of HPA axis activity and GSH-homeostasis. Lastly, (5) we investigated whether markers of the HPA axis and the GSH-antioxidant balance were associated with cerebral white matter microstructure in a sex-dependent manner. Given the extended literature reporting the influence of pubertal hormones on HPA-axis [49], redox regulation [50, 51] and brain maturation [11], we explored in a secondary hypothesis whether the aforementioned associations were related to pubertal status and gonadal hormones (estradiol and testosterone).

## Methods

### Subjects

This study was nested in the Mindfulteen study, which is a randomized controlled cross-over clinical trial investigating the impact of a stress-reduction intervention based on mindfulness meditation on adolescent well-being [52]. Briefly, the Mindfulteen study recruited adolescents between 13 and 15 years old. They were excluded if they had a chronic somatic disease or a significant medical condition, if they benefited from psychotherapy in the last 6 months, if they received a psychotropic medication in the past month and if they met criteria for any psychiatric disorder of the DSM-IV except for a current anxiety disorder and/or a past (>6 months) episode of major depressive disorder. The study protocol was approved by the Geneva Regional Ethical Committee (CCER 2018–01731). For the current study, we included only data drawn from the baseline evaluation and blood sampling (Visit (V)0 and V1), prior to the intervention. Detailed information concerning recruitment, inclusion and exclusion criteria are available in the published protocol [52].

### Clinical evaluation

Participants underwent a thorough psychiatric evaluation by trained psychologists using the K-SADS [53] during the baseline evaluation (V0). Trait anxiety was assessed using the State and Trait Anxiety Inventory for Children (STAIC-T) [54] and depressive symptoms were assessed with the Beck Depression Inventory (BDI) [55]. Information on the pubertal status (PS) was drawn from the K-SADS’ item assessing attainment of puberty (based on the apparition of menarche in females and changes in voice and pilosity in males). One male did not provide information on the PS.

### Blood analysis

Blood was collected within 7 days before or after the MRI (V1), in the morning (between 7 and 8.30 a.m.) after an overnight fast. Enzymatic activity of GPx and Gred were assessed in red blood cell lysates using an in-house protocol described in the Supplementary Information. Levels of reduced GSH were measured in red blood cell lysates using a diagnostic kit (Calbiochem) and normalized to blood volume (see Supplementary Information). Steroids were measured in the serum by liquid chromatography-tandem mass spectrometry (LC-MS/MS) in the laboratory of Clinical Chemistry of the Lausanne University Hospital. Gonadal steroids of interest included levels of estradiol (E2) and testosterone (T). We also focused on glucocorticoids directly involved in cortisol synthesis and metabolism, namely 11-deoxycortisol, cortisol and cortisone, as well as the ratio between cortisol and its precursor and metabolite (cortisol/11-deoxycortisol and cortisol/cortisone) (Fig. 3F).

One female subject missed the blood test. One male subject had an extreme Gred activity value (beyond 3 SD of the mean) which was considered as a technical outlier and was removed from all analyses involving Gred activity. Only one female subject was taking the contraceptive pill at the time of the first visit and was removed from the analysis involving steroids due to cortisol value >2 SD. This resulted in 67 subjects with measures of GSH markers, 66 subjects with measures of steroids and a total 65 subjects with measures of GSH markers and steroids.

### Image acquisition

Participants underwent a brain scan on a 3T Magnetom TIM Trio scanner (Siemens, Germany) equipped with a 32-channel head coil. Each scanning session included a magnetization-prepared rapid acquisition gradient echo (MPRAGE) T1-weighted sequence with 1 mm in-plane resolution and 1 mm slice thickness and a diffusion spectrum imaging (DSI) sequence including 128 diffusion-weighted images with a maximum *b*-value of 8000 s/mm^2^ and one b0 reference image. Detailed specifications of each sequence are provided in the Supplementary Information.

### Generalized fractional anisotropy computation

QUAD (QUality Assessment for DMRI) was used to quantitatively assess the quality of the diffusion-weighted images and extract different quality control metrics for each subject [56]. This tool computes the quality control metrics using the results provided by the EDDY tool after correcting each individual dMRI for motion and induced currents. Total outliers, average absolute motion, average relative motion, signal-to noise ratio and contrast-to-noise ratio were used to assess the quality control of the DWIs. An automatic image correction and processing workflow was applied over the individual diffusion-weighted images. Briefly, the workflow employed mrtrix (v.3.0.3) [57] and FSL (v.6.0.3) [58] for performing the following correction steps: denoising, bias correction, intensity normalization, head motion correction (with gradient table rotation), eddy current and distortion correction. A registration-based approach using Advanced Normalization Tools (ANTs, v.2.4.1) [59] was implemented to correct the geometrical distortion along the phase-encoding direction. Dipy (v.1.5.0) [60] was applied over the corrected DWIs to fit both second order tensors and intravoxel orientation distribution functions (ODF) via the Simple Harmonic Oscillator-based Reconstruction and Estimation method (SHORE) [61]. The resulting ODFs derived from the corrected DWIs were employed to compute the generalized fractional anisotropy (gFA). Similar to FA, gFA values range from 0 to 1, indicating zero to maximal orientational anisotropy in the ODF. Each individual gFA map was inspected manually for gross abnormalities and/or artifacts. Additional information on the generation of gFA maps is provided in the Supplementary Data.

### Statistical analysis

Statistical analysis was run using R v. 4.0.5. We examined gender differences in demographic and clinical characteristics using *t*-test for continuous outcomes and chi-square analysis for categorical outcomes. When normality was not assumed, permutation tests were performed using 5000 random permutations. Standard ordinary least square (OLS) regressions were used to investigate sex-differences in GSH-metabolizing enzymes, GSH levels and adrenal steroids while adjusting for age and Body Mass Index (BMI), based on the reported influence of these covariates on the activity of the GSH-system enzymes and steroid levels [62, 63]. Anxiety and depression scores were added in the OLS model to explore the association between GSH-homeostasis, steroid levels and internalizing symptoms. As females and males differed significantly in the levels of the main outcomes (internalizing symptoms, glucocorticoid levels and GPx activity), we added a sex-interaction term to explore sex-specific associations. In secondary analyses, we added PS and the levels of gonadal hormones (E2 and T) as covariates to explore the independent effect of pubertal maturation and gonadal hormones. Spearman correlation was used to investigate the relation between the activity of the two enzymes GPx and Gred in boys and girls separately. Statistical difference between the two independent correlation coefficients was calculated using the Fisher test. For all statistical analyses, the effect was considered significant at a threshold of *p* value < 0.05. Causal mediation analysis was performed using the “mediation” package in R. As a prerequisite, mediation analysis was run only when both the presupposed mediator and independent variable were significantly associated with the dependent variable. Sex, age, and BMI were entered as covariates in each analysis. Confidence intervals of the estimated mediation effect was inferred following non-parametric bootstrap resampling with 5000 simulations. Causal mediation effect was considered when the 95% confidence interval did not include 0.

### Image analyses

Associations between blood markers and white matter microstructure (gFA) were tested across the entire brain white matter volume. Whole brain voxel-based-analysis (VBA) in white matter was performed for each sex separately on the diffusion images-derived gFA maps using generalized linear models, corrected for family-wise error rate using Threshold-Free Cluster Enhancement as implemented in FSL v.6.0.3 software package. Linear models included age as covariate.

## Results

### Demographics

A table recapitulating demographics according to sex is displayed in Table 1. Briefly, the sample comprised 68 adolescents of whom 39 were females (57.4%) and 29 were males (42.6%). Sex groups did not differ in age, BMI, pubertal status, and tobacco use. Ten adolescents (14.7%) met the criteria for a current anxiety disorder, of whom 3 boys and 6 girls had a diagnosis of generalized anxiety disorder and 1 girl had a diagnosis of panic disorder. Twelve adolescents (18%) had a history of previous major depressive episode.

**Table 1:** Demographics by sex group.

	Females (*N* = 39)	Males (*N* = 29)	*p* value
Age (years)			
Mean (SD)	14 (±0.91)	14 (±0.91)	0.43
Body mass index (kg/m^2^)			
Mean (SD)	20 (±2.6)	20 (±3.0)	0.42
Tobacco use			
No	38 (97%)	27 (93%)	0.79
Yes	1 (3%)	2 (7%)	
Pubertal status (PS)			
Pre-puberty	10 (26%)	11 (38%)	0.36
Post-puberty	29 (74%)	17 (59%)	
Missing	0 (0%)	1 (3.4%)	
Panic disorder			
None	36 (92%)	28 (97%)	0.64
Sub-clinical	2 (5%)	1 (3%)	
Clinical	1 (3%)	0 (0%)	
Generalized anxiety disorder			
None	32 (82%)	25 (86%)	0.82
Sub-clinical	1 (3%)	1 (3%)	
Clinical	6 (15%)	3 (10%)	
Previous major depressive disorder			
None	32 (82%)	24 (83%)	1
Previous major depressive episode	7 (18%)	5 (17%)	

### Internalizing symptoms

**Fig. 1:**
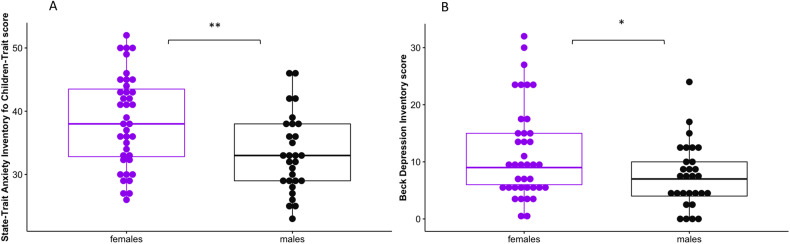
Effect of sex on internalizing symptoms. Female adolescents display significantly higher scores of anxiety (State-Trait Anxiety Inventory for Children Trait (STAIC-T)) (**A**) and depression (Beck Depression Inventory (BDI)) (**B**) than males. *p < 0.05, **p < 0.01.

Adolescent females were significantly more anxious (Mean = 38.5, SD = 7.28) than males (Mean = 33.2, SD = 6.12) according to the STAIC-T (*t*(66) = 3.14, *p* = 0.003) and more depressed than males according to the BDI (females: median = 9, interquartile range = 9; males: median = 7, interquartile range = 6, *p* = 0.021) (Fig. 1).

### GSH-redox cycle system

**Fig. 2:**
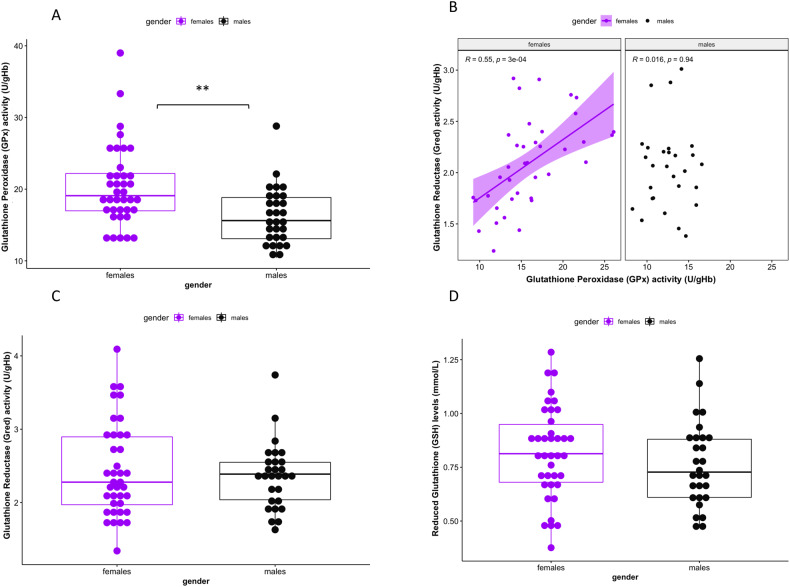
Effect of sex on glutathione redox cycle regulation. Females display higher glutathione peroxidase (GPx) activity compared to males (**A**) and a strong correlation between GPx and glutathione reductase (Gred) activities (*r* = 0.55, *p* < 0.001) compared to males in which no correlation is found (**B**). The two correlation coefficients are significantly different (Fisher test *p* < 0.05). No sex-differences are observed for Gred activity (**C**) and levels of reduced glutathione (GSH) (**D**).

Regarding sex-differences in GSH-redox cycle system, GPx activity was higher in erythrocytes of females than males (*ß* = 4.09545, *p* = 0.001) (Fig. 2). Gred activity and levels of reduced GSH did not differ between sexes. Secondary analyses indicated that GPx and Gred activities were higher in post-pubertal compared to pre-pubertal adolescents (respectively: *ß* = 4.22081, *p* = 0.012; *ß* = 0.432078, *p* = 0.037) but were not associated with gonadal hormones. As the measure of the relation between activity of complementary enzymes GPx and Gred may reflect GSH system redox status, we looked at the correlation between GPx and Gred activity in each sex. In females, there was a positive correlation between GPx and Gred activities (*r* = 0.55, *p* < 0.001), indicating a highly regulated oxidoreductive balance of the GSH-redox cycle, whereas in males GPx and Gred did not correlate (*r* = 0.016, *p* = 0.940). The two correlation coefficients were significantly different (two-tailed Fisher test, *z* = 2.341, *p* = 0.021).

### HPA-axis markers

**Fig. 3:**
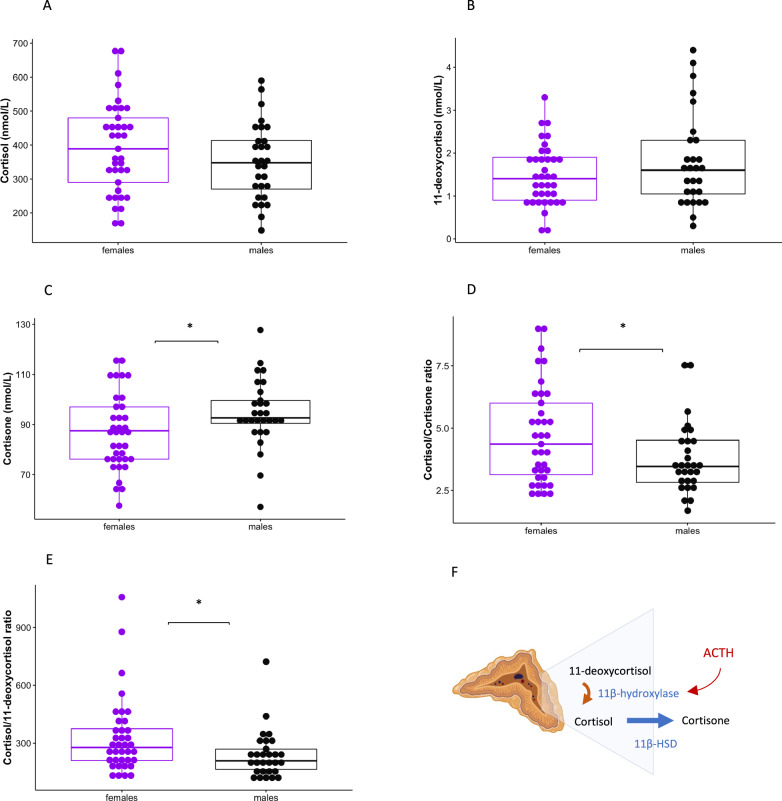
Sex-differences in cortisol metabolism. No differences in basal serum cortisol (**A**) and 11-deoxycortisol (**B**) between males and females. Females show lower levels of cortisone (**C**) and higher levels of cortisol/cortisone ratio (**D**) and cortisol/11-deoxycortisol ratio (**E**) than males. **F** 11β-hydroxylase catalyzes the conversion of 11-deoxycortisol to cortisol in the adrenal cortex, under the influence of adrenocorticotropic hormone (ACTH). Cortisol is converted to its inactive metabolite cortisone in peripheral tissues by the 11β-hydroxysteroid-dehydrogenase type II. Image created in Biorender.com. *p value <0.05.

To assess whether the sex-difference in internalizing symptoms was associated with a difference in HPA-axis related adrenal steroids, we first compared the basal levels of glucocorticoids (11-deoxycortisol, cortisol, cortisone) involved in cortisol metabolism as well as relevant steroid ratios (i.e., reflecting indirect activity of key enzymes), between males and females. Significant sex-differences were found for cortisone with lower levels in females compared to males (*ß* = −7.04832, *p* = 0.045), as well as higher cortisol/11-deoxycortisol ratio (*ß* = 89.302, *p* = 0.041) and cortisol/cortisone ratio (*ß* = 0.871113, *p* = 0.043) in females compared to males (Fig. 3). No difference was found for levels of cortisol, nor 11-deoxycortisol. There was no effect of PS on glucocorticoid levels, however a positive association was found between cortisone and testosterone (*β* = 1.5991, *p* = 0.002), and a negative association between cortisol/11-deoxycortisol and testosterone (*β* = −15.780, *p* = 0.015).

### Association between internalizing symptoms and the GSH-antioxidant balance

**Fig. 4:**
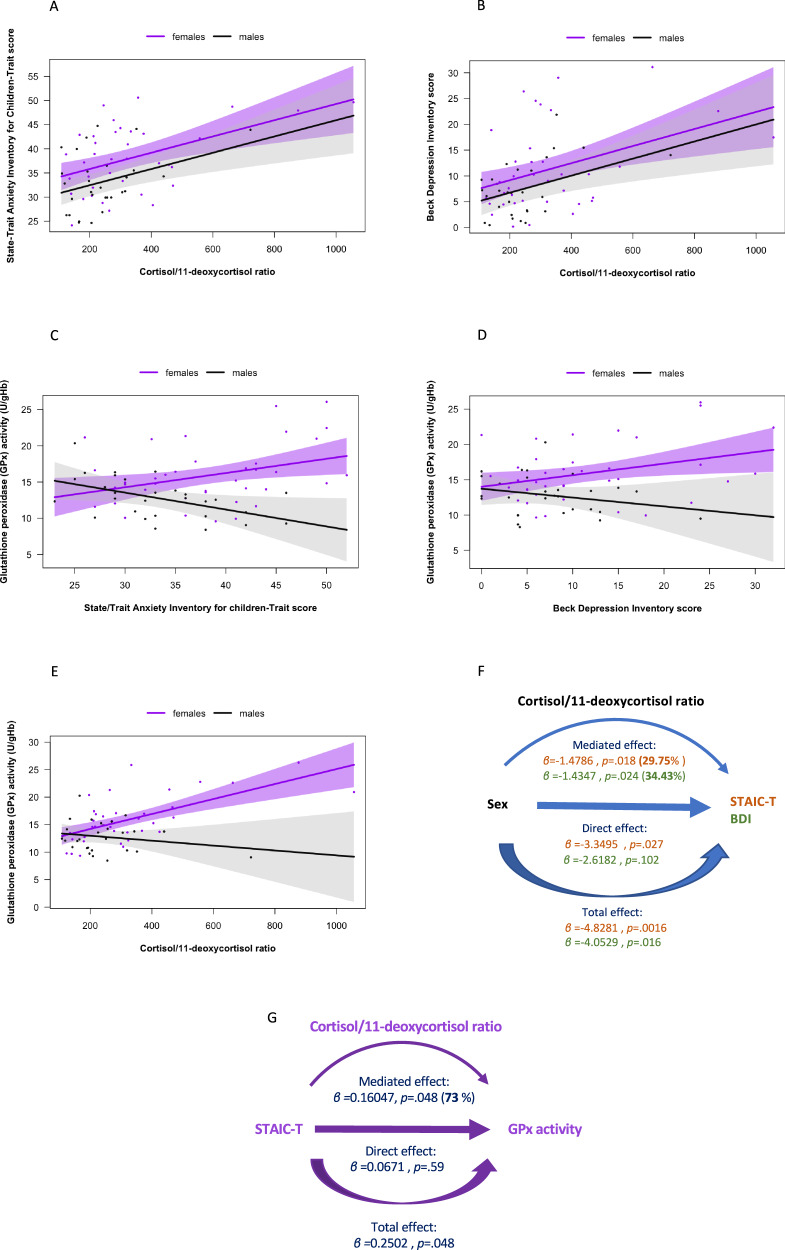
Association between cortisol/11-deoxycortisol ratio, GSH-redox cycle homeostasis, internalizing symptoms and HPA-axis function. Ordinary least square (OLS) regression showing a positive association between cortisol/11-deoxycortisol ratio and State-Trait Anxiety Inventory Trait (STAIC-T) scores (**A**) (*p* < 0.001) and BDI (**B**) (*p* < 0.01) scores while controlling for sex, age and body mass index (BMI). Regression lines are displayed by sex. Sex-by-trait anxiety (STAIC-T) (*p* < 0.01) (**C**), sex-by-depression (BDI) (*p* < 0.05) (**D**) and sex-bycortisol/11-deoxycortisol (*p* < 0.01) (**E**) interaction in association with GPx activity. In females, significant positive association between STAI-T and GPx activity (*p* < 0.05) (**C**), BDI and GPx activity (*p* < 0.05) (**D**) and cortisol/11-deoxycortisol ratio and GPx activity (*p* < 0.0001) (**E**). In males, negative association between STAI-T and GPx activity (*p* < 0.05) (**C**). **F** Mediation of the cortisol/11-deoxycortisol ratio on sex-differences in STAIC-T and Beck Depression Inventory (BDI) scores using causal mediation analysis. **G** Mediation of cortisol/11-deoxycortisol ratio on the relationship between STAI-T and GPx activity in females using causal mediation analysis.

We next investigated whether levels of trait anxiety (STAI-T) and depression (BDI), which were higher in females than in males, were associated with markers of GSH-redox cycle. GSH levels as well as GPx and Gred activities were regressed on STAI-T and BDI with sex, BMI and age as covariates. Further, sex was added as an interaction term to investigate sex-specific associations. No significant association between STAI-T and GPx or BDI and GPx were found in the whole cohort. However, there was a significant STAI-T by sex interaction (*F*(5,61) = 4.596, *p* = 0.002) as well as significant BDI by sex interaction (*F*(5,61) = 3.959, *p* = 0.026) in predicting GPx activity (Fig. 4). Simple slopes indicated that in females, GPx activity was positively associated with STAI-T (*ß* = 0.2415, *p* = 0.031) and BDI (*ß* = 0.23509, *p* = 0.017) whereas in males GPx activity was negatively associated with STAI-T (*ß* = −0.3441, *p* = 0.026) and not significantly associated with BDI (*ß* = −0.20981, *p* = 0.225). No significant association between internalizing symptoms and Gred activity nor GSH levels were found neither in the whole cohort nor in a sex-specific way.

### Associations between internalizing symptoms and HPA-axis markers

To investigate if the sex-difference observed for cortisone, cortisol/11-deoxycortisol ratio and cortisol/cortisone ratio was related to the higher internalizing symptoms observed in females than males, we next regressed STAIC-T and BDI scores on the glucocorticoids and glucocorticoid ratios of interest. Sex was first added as covariate and then as an interaction term to investigate sex-specific associations. The cortisol/11-deoxycortisol ratio was strongly associated with STAI-T (*ß* *=* 0.0168*, p* < 0.001) and BDI (*ß* = 0.0165*, p* = 0.002) in the whole cohort (Fig. 4A, B). No sex by cortisol/11-deoxycortisol ratio interaction was found, suggesting that internalizing symptoms are positively associated with the cortisol/11-deoxycortisol ratio in both sexes. No other significant association was found between glucocorticoid levels/ratio (i.e., cortisol, cortisone, 11-deoxycortisol, cortisol/cortisone ratio) and internalizing symptoms (i.e., STAI-T and BDI) in the whole cohort. To address whether the sex-difference in internalizing symptoms was mediated by differences in the cortisol/11-deoxycortisol, we conducted causal mediation analysis. STAI-T and BDI were entered in a regression model as dependent variables with sex, age, BMI and Cortisol/11-deoxycortisol ratio as independent variables. Sex was entered as the treatment of the mediation and Cortisol/11-deoxycortisol ratio as the mediator. Cortisol/11-deoxycortisol ratio accounted for 29.75% (*p* = 0.018) of the total effect of sex on STAI-T, and 34.43% (*p* = 0.024) of the total effect of sex on BDI (Fig. 4C).

### Associations between GSH-antioxidant system and the HPA-axis

Following the hypothesis of a biological interaction between the GSH system and the HPA axis, we further explored how the cortisol/11-deoxycortisol ratio related to GPx activity and if the association was sex-specific. Cortisol/11-deoxycortisol ratio was significantly positively associated with GPx activity in the whole cohort (*ß* = 0.011254, *p* = 0.002). Moreover, there was a significant sex by Cortisol/11-deoxycortisol ratio interaction (*F*(5,60) = 6.236, *p* = 0.0011) (Fig. 4C). Cortisol/11-deoxycortisol ratio levels were positively associated with GPx activity in females (*ß* = 0.0160756*, p* < 0.0001) but not in males. Given the sex-specificity of this association, we next evaluated in females the directionality of the association between cortisol/11-deoxycortisol ratio, GPx activity and internalizing symptoms using causal mediation analysis: GPx did not mediate the association between cortisol/11-deoxycortisol ratio and STAI-T nor BDI, whereas the cortisol/11-deoxycortisol accounted for 73% (*p* = 0.048**)** of the association between STAI-T and GPx activity in females (Fig. 4D). The mediated effect of Cortisol/11-deoxycortisol ratio on the association between BDI and GPx activity did not reach significance (*p* = 0.109).

### Association with white matter diffusion properties

**Fig. 5:**
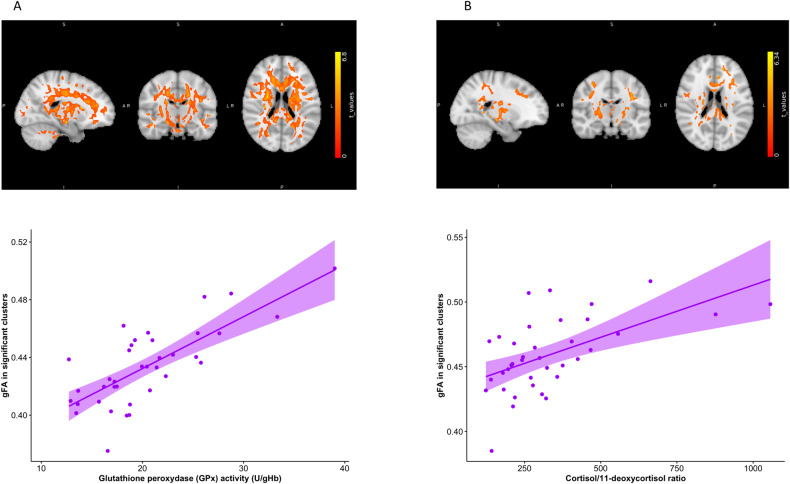
Correlation between white matter microstructure and markers of GSH-redox cycle homeostasis and HPA-axis function in females. Widespread correlation in female subjects between (**A**) generalized fractional anisotropy (gFA) and glutathione peroxidase (GPx) activity, (**B**) gFA and cortisol/11-deoxycortisol ratio. All voxels in the cluster are significant at the whole brain level (non-parametric family-wise error corrected *p* < 0.05). Scatterplots display correlation between (**A**) GPx activity and (**B**) cortisol/11-deoxycortisol ratio on *x*-axis and mean gFA values in significant clusters on *y*-axis.

Lastly, we investigated if higher levels of anxiety and depression in females and specific markers of the HPA-axis (cortisol/11-deoxycortisol ratio) and GSH-antioxidant balance (GPx) were related to alterations in brain white matter microstructure. We first ran a whole brain voxel-based analysis (VBA) in white matter looking at the association between regional gFA and STAI-T as well as between gFA and BDI scores. There was no white matter region showing a significant association between gFA and STAI-T or BDI, nor any sex effect. Next, VBA was conducted to estimate associations between gFA and both GPx and the cortisol/11-deoxycortisol ratio. In females, GPx was strongly and positively associated with gFA in widespread regions of white matter (Fig. 5). No association was found in males. Similarly, cortisol/11-deoxycortisol ratio was positively associated with widespread clusters in females but not in males. When cortisol/11-deoxycortisol ratio and GPx were added conjointly in the model, the significant association with cortisol/11-deoxycortisol was lost, whereas important clusters remained significant with GPx, suggesting that GPx activity mediated the association between cortisol/11-deoxycortisol ratio and gFA.

## Discussion

In this study, we aimed to investigate the sex-specific associations between internalizing symptoms, GSH-redox cycle regulation and HPA-axis function during the peripubertal period. In young adolescents from the general population, we found increased anxiety and depressive symptoms, increased GSH-redox cycle regulation (e.g., increased GPx activity, strong correlation between GPx and Gred activity) as well as increased HPA-axis activity (e.g., increased cortisol/11-deoxycortisol) in females as compared to males. Increased GPx activity and cortisol/11-deoxycortisol ratio were not only associated with high scores of internalizing symptoms, but also with a widespread increase in white matter gFA in females’ brain only. Mediation analysis indicated a causal effect of the cortisol/11-deoxycortisol ratio on the sex-difference in internalizing symptoms. Moreover, the cortisol/11-deoxycortisol ratio mediated the association between anxiety and GPx activity in females, suggesting a causal role of HPA-axis function in mediating changes in redox homeostasis associated with anxiety. Lastly, changes in GPx activity appeared to account for the association between cortisol/11-deoxycortisol and gFA in females. Altogether, our results suggest the involvement of tight and sex-specific interactions between GSH-redox cycle homeostasis and the HPA-axis, in the occurrence of internalizing symptoms and maturation of brain white matter in adolescent females.

Higher levels of internalizing symptoms in female compared to male adolescents recapitulated well known epidemiological findings [6, 8]. Based on these results, we further explored the biological mechanisms that may underlie this sex-difference in internalizing symptoms. The HPA-axis matures during adolescence and its activity can be quantified by the blood measurement of several glucocorticoids related to the metabolism of cortisol, the main stress hormone. Importantly, the ratio of such glucocorticoids may reflect the activity of enzymes involved in cortisol metabolism and which are under the influence of the adrenocorticotropic hormone (ACTH) [64]. Although few studies have characterized the adrenal steroid profile of healthy adolescents and its changes throughout puberty, some have underlined the high specificity of some steroids as well as their ratio in depression, especially in adolescents [42, 43]. For example, basal corticosterone levels and the ratio of corticosterone to its precursor 11-deoxycorticosterone, indicative of 11β-hydroxylase activity, were reported to be increased in adolescent depression, with more discriminant features in girls [65]. In the present cohort of adolescents, we found that females exhibited a higher cortisol/11-deoxycortisol ratio than males, a difference that mediated in part the higher levels of internalizing symptoms observed in females, suggesting a causal effect of HPA-axis function on internalizing symptoms. 11β-hydroxylase catalyzes the conversion of 11-deoxycortisol to cortisol and is influenced by ACTH, therefore increased cortisol/11-deoxycortisol ratio may involve a higher ACTH drive and reflect heightened activity of the HPA-axis. In this regard, increased cortisol/11-deoxycortisol ratio was reported in depressed subjects following dexamethasone suppression test [66]. Genetic polymorphism in the 11β-Hydroxylase gene (CYP11B1) have been also found to be associated with increased risk for late-life depression in women specifically [67]. However, the cortisol/11-deoyxcortisol ratio should not be interpreted as a sole result of 11β-Hydroxylase activity, as it may be influenced by changes in the downstream metabolism of cortisol, as well as alterations in the levels of corticosteroid binding globulin (CBG), which may increase levels of serum bound (inactive) cortisol and affect levels of total cortisol measured in serum. To our knowledge, our study is the first to explore sex-specific associations between the cortisol/11-deoxycortisol ratio and dimensional symptoms of anxiety and depression in adolescents. In line with prospective studies, these results highlight the function of the HPA-axis during the peripubertal period as a possible factor associated with greater vulnerability to internalizing symptoms [13] and emphasizes that this vulnerability may be exacerbated in females due to the emergence of sexually dimorphic HPA-axis regulation [68, 69].

In addition to HPA axis maturation, sexual dimorphism of the GSH metabolism and GSH-dependent responses have been reported [70] and may also be involved in sex-differences regarding internalizing symptoms. Accordingly, we found higher GPx activity in female adolescents as compared to males. This result is in line with previous studies which reported higher activity of GPx in pre-menopausal women compared to men, as well as in menopaused women under hormone replacement therapy, and in adolescent females compared to males [71, 72]. Importantly, females showed a strong correlation between GPx and Gred activities that may indicate a highly regulated oxidoreductive balance of the GSH-redox cycle during adolescence compared to males. Moreover, the female-specific association between GPx activity and anxiety, which appears mediated by the cortisol/11-deoxycortisol ratio, suggests a time and sex-specific interplay between HPA-axis function, GSH-antioxidant balance and the expression of internalizing symptoms. Based on experimental studies in preclinical models, two main hypotheses can be drawn. First, changes in the redox status occurring during the pubertal transition may show sex-specific patterns, as the rising gonadal hormones have known effects on the redox balance [50]. This change in redox status may in turn affect the synthesis of steroid hormones, as adrenal enzymes involved in steroid synthesis are highly sensitive to the redox state and the supply of oxidoreductive couples provided in great part by the GSH-system, such as NADP+/NADPH [73, 74]. The importance of a finely tuned redox balance required for proper HPA-axis function is further stressed in animal experiments, in which antioxidant treatment abolished the increased HPA-axis activity observed following chronic stress, but also promoted a hyperactivation of the HPA-axis activity in non-stressed animals [37]. Second, the increase in HPA-axis sensitivity and reactivity occurring during adolescence may promote an increase in metabolic demand and the generation of ROS, leading to a compensatory increase in the activity of detoxifying enzymes in females [25]. This hypothesis is supported by the causal mediation analysis, suggesting a causal role of HPA-axis function on changes of redox homeostasis in females. Consistently, experiments in rats show sex-specific antioxidant response to stress in the brain, with females showing an increased GPx activity compared to males [75], indicating sex-specific redox adaptations following stress exposure. Clinical studies report increased GPx activity in erythrocytes of patients with social phobia [34] and panic disorder [76]. Increased GPx activity was also reported in major depression [33] but with contrasting findings of decreased activity in another study [77]. Increased GPx activity may therefore represent compensatory mechanisms and may be influenced by sex, age, and the stage of illness. Together, our results suggest a tight interaction between stress, activity of the HPA-axis and redox homeostasis in females, that may underlie their increased vulnerability to internalizing disorders during the adolescent period.

In this study, we found no association between gFA and internalizing symptoms (i.e., STAI-T and BDI). While previous studies reported alterations in white matter microstructure in association with anxiety and depression self-ratings [78–80], we find this result not surprising given that this sample included adolescents from the general population with overall moderate levels of internalizing symptoms, as only ten adolescents met the criteria for a clinical anxiety disorder. On the other hand, our results indicate that increased GPx activity was specifically associated with widespread patterns of increased gFA in females and accounted for the association between cortisol/11-deoxycortisol ratio and gFA. While no previous study investigated the association between peripheral GSH-redox cycle regulation and white matter microstructure, our results, indicating higher integrity of white matter microstructure in association with increased peripheral antioxidant defenses in females, are in line with studies reporting a positive association between levels of brain GSH and FA in young adults [42, 48]. Oligodendrocytes have a high metabolism to build and maintain the myelin sheets around the axons [81] as well as high levels of iron necessary for some enzymes involved in the synthesis of myelin [82]. Therefore, these glial cells are particularly vulnerable to OxS and need to keep an appropriate redox homeostasis via the antioxidant systems, including the GSH redox cycle. Notably, Corcoba et al. [83] showed reduced FA along some white matter tracts (including the fornix) in mice with a weak GSH synthesis capacity. Interestingly, while oxidative stress can affect the viability of oligodendrocytes, an oxidized redox state can on the other hand favor the differentiation/maturation of oligodendrocytes [84, 85]. Therefore, further studies on redox modulating factors impacting myelin microstructure during development is needed. Last, developmental studies investigating the effects of chronic stress converge on evidence that stress may impact brain maturational trajectory and rate, especially accelerating the maturation of affective-related circuits [86, 87], which may both represent an advantage in adverse conditions but may also limit plasticity and raise vulnerability to subsequent anxiety and affective disorders. Accordingly, stress is associated with faster pubertal maturation [88] and precocious puberty increases the risk for internalizing disorders [89, 90]. Altogether, these results suggest that changes in the redox status associated with altered HPA-axis function and internalizing symptoms may play a role in the modulation of white matter microstructure maturation.

The main limitation of the study is the relatively low number of participants when stratified by sex. The results obtained must be interpreted with caution and should be replicated in a wider population of adolescents. Although all analyses investigating sex-differences were adjusted for internalizing symptoms, sex-specific findings may be influenced by the important difference in baseline levels of anxiety and depression levels between males and females in this cohort. Additionally, a main limitation is the lack of pubertal assessment by Tanner-staging, which allows a finer definition of the pubertal transition. Nevertheless, we reinforced the accuracy of the pubertal status estimation by adding T and E2 levels as covariates in the secondary analysis. Another main limitation is the fact that only the blood antioxidant system of GPx/Gred activity couple and GSH levels were analyzed, which may not reflect brain GSH-antioxidant regulation. Similarly, while the cortisol/11-deoxycortisol may reflect ACTH tone, it is not yet established how this ratio relates to HPA-axis reactivity, which would require a dynamic measure of cortisol secretion assessed over serial blood samples. Finally, the correlational nature of these findings, as well as the cross-sectional design used to conduct mediation analyses prevent from drawing any strong causal biological inference. Temporal contingency between these constructs should be investigated in longitudinal settings, considering important factors influencing HPA-axis activity such as adverse life events.

To conclude, these results highlight important interactions between HPA-axis and redox status as well as their potential role in the maturational trajectory of white matter microstructure. It lends support to the hypothesis that sexual dimorphism in HPA-axis and redox regulation may underly sex-differences in vulnerability to internalizing disorders. Longitudinal studies are warranted to investigate the association between peripheral redox status and brain maturation throughout the adolescent transition.

### Supplementary information

Supplementary

## Data Availability

The dataset analyzed in the current study is available from the corresponding author upon reasonable request.
