# Supplementary material for: Sex-specific interactions between stress axis and redox balance are associated with internalizing symptoms and brain white matter microstructure in adolescents
Source: Transl Psychiatry. 2024 Jan 17;14:30. doi: 10.1038/s41398-023-02728-4 (PMC10794182; doi:10.1038/s41398-023-02728-4)
Supplement: Supplementary file 1 — Supplementary [file 41398_2023_2728_MOESM1_ESM.pdf]

Supplementary material

*Blood analysis*

Blood was collected within seven days before or after the MRI (V1), in the morning (between 7 and 8.30 am) after an overnight fast. EDTA and dry tubes were kept on ice during blood preparation and the different fractions were stored at -80°. EDTA tubes were centrifugated at 3000 g for 5 minutes at 4°C, plasma was removed, and erythrocytes were washed twice in cold NaCl 0,9% before being flash frozen at -80° until their analysis. Levels of reduced glutathione (GSH), glutathione peroxidase (GPx) and glutathione reductase (GR) activities were measured in red blood cells. GR activity was assessed in 8 µL of hemolyzed blood cells incubated in a 200 µl phosphate buffer solution (100mM, pH 7.5) containing EDTA (0.6mM), and non-limiting levels of oxidized glutathione (GSSG, 2.5mM) and NADPH (0.25mM). The activity of GR (1) was expressed as the amount of NADPH used by GR (in nmole/min at 22°C) to reduce GSSG. The decrease of NADPH per min was measured using the decrease of absorption at 340 nm (per min) and quantified using the NADPH 340 nm absorption coefficient. GPx activity was assessed in 8 µL of hemolyzed blood cells incubated in a 200 µl phosphate buffer solution (100mM, pH 7.5) with EDTA (0.6mM), and non-limiting amounts of GSH (2.5mM), NADPH (0.25mM), GR (0.84U/ml; Fluka) and tert-butyl hydroperoxide (TBHP, 0.8mM, Fluka). The activity of GPx(2) is proportional to the amount of NADPH used to reduce the GSSG produced during the reduction of TBHP by GPx. We expressed GPx activity as the amount of NADPH used (in nmole/min at 22°C). As for GR activity, the decrease of NADPH per min was measured using the decrease of absorption at 340 nm (per min) and quantified using the NADPH 340 nm absorption coefficient. Both GPx and GR activities were normalized to the amount of hemoglobin. The average inter-assay coefficient variation for GPx and GR were respectively 3.4 and 5.8, while the mean inter-assay reproducibility (expressed as SEM in

%) for GPx and GR were respectively 3.6% and 6.2%. The amount of GSH in red blood cells was measured using a spectrophotometric assay kit (Calbiochem GSH assay, ref. 354102, Merckmillipore) according to the manufacturer instructions (inter and extra-assay reproductibiliy: SEM in % < 2%) and expressed as mmole GSH / L blood.

*Image acquisition*

Participants underwent a brain scan on a 3T Magnetom TIM Trio scanner (Siemens, Germany) equipped with a 32-channel head coil. Each scanning session includes a magnetization-prepared rapid acquisition gradient echo (MPRAGE) T1-weighted sequence with 1 mm in-plane resolution and 1 mm slice thickness, covering 240 × 257 × 160 voxels. The repetition (TR), echo (TE), and inversion (TI) times were 2300, 2.98, and 900 ms, respectively. The diffusion spectrum imaging (DSI) sequence included 128 diffusion-weighted images with a maximum b-value of 8000 s / mm2 and one b0 reference image. The acquisition volume was made of 96 × 96 × 34 voxels with 2.2 × 2.2 × 3 mm resolution. TR and TE were 6800 and 144 ms, respectively.

*Skull-stripping*

All the individual T1w images were processed where subject-specific brain masks were computed using the graph-cut based skull-stripping algorithm implemented in the Computational Anatomy Toolbox (*CAT12*). The skull-stripped T1w images were binarized, visually inspected and manually corrected, if necessary, for possible brain segmentation errors.

*Generalized fraction anisotropy computation*

An automatic image correction and processing workflow was applied over the individual diffusion-weighted images. Briefly, the workflow employed Mrtrix3 (v. 3.0.3 )(3) and FSL (v.

6.0.3)(4) for performing the following correction steps: denoising, bias correction, intensity normalization, head motion correction (with gradient table rotation), eddy current and distortion correction. A registration-based approach using Advanced Normalization Tools (ANTs, v. 2.4.1) (5) was implemented to correct the geometrical distortion along the phase-encoding direction. Dipy (v1.5.0)(6) was applied over the corrected DWIs to fit both second order tensors and intravoxel orientation distribution functions (*ODF*) via the Simple Harmonic Oscillator-based Reconstruction and Estimation method (*SHORE*) (7). On one hand, the diffusion tensors were used to compute different voxel-wise scalar maps such as fractional anisotropy (*FA*) and mean diffusivity (*MD*). On the other hand, SHORE tackles the limitation of FA and MD values in white matter regions with more complex organization (fiber crossings, twisting or kissing) because it creates ODFs with higher angular resolution than the standard diffusion tensor or DSI reconstructions. The resulting ODFs were employed to compute the generalized fractional anisotropy (g*FA*). Similar to FA, gFA values range from 0 to 1, indicating zero to maximal orientational anisotropy in the ODF.


1. Long WK, Carson PE. Increased erythrocyte glutathione reductase activity in diabetes mellitus. Biochem Biophys Res Commun. 1961 Aug 23;5(5):394–9.
2. Günzler WA, Kremers H, Flohé L. An Improved Coupled Test Procedure for Glutathione Poroxidase (EC 1.11.1.9.) in Blood. Clinical Chemistry and Laboratory Medicine. 1974 Jan 1;12(10):444–8.
3. Tournier JD, Smith R, Raffelt D, Tabbara R, Dhollander T, Pietsch M, et al. MRtrix3: A fast, flexible and open software framework for medical image processing and visualisation. NeuroImage. 2019 Nov 15;202.

4. Jenkinson M, Beckmann CF, Behrens TEJ, Woolrich MW, Smith SM. FSL. NeuroImage. 2012 Aug;62(2):782–90.

5. Avants BB, Tustison NJ, Stauffer M, Song G, Wu B, Gee JC. The Insight ToolKit image registration framework. Frontiers in neuroinformatics. 2014 Apr 28;8(APR).

6. Garyfallidis E, Brett M, Amirbekian B, Rokem A, van der Walt S, Descoteaux M, et al. Dipy, a library for the analysis of diffusion MRI data. Frontiers in neuroinformatics. 2014 Feb 21;8(FEB).

7. Ozarslan E, Guan Koay C, Basser PJ. Simple harmonic oscillator based reconstruction and estimation for one-dimensional q-space magnetic resonance (1D-SHORE).

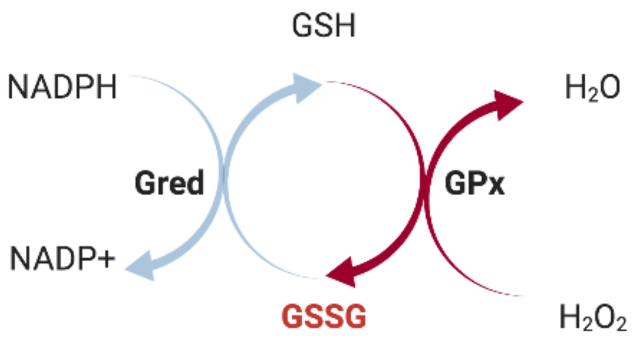


Supplementary Figure S1: Overview of glutathione metabolizing enzymes

*Hydrogen peroxide ($H_2O_2$) can be reduced by the glutathione peroxidase (GPx). GPx couples the reduction of $H_2O_2$ with the oxidation of glutathione (GSH). Oxidized glutathione (GSSG) is further reduced by GSH reductase (Gred) in a NADPH-dependent manner. Image created in* [Biorender.com](Biorender.com)
